# Teachers' Knowledge and Stigmatizing Attitudes Associated With Obsessive-Compulsive Disorder: Effectiveness of a Brief Educational Intervention

**DOI:** 10.3389/fpsyt.2021.677567

**Published:** 2021-06-02

**Authors:** Antonio Chaves, Sandra Arnáez, María Roncero, Gemma García-Soriano

**Affiliations:** Departamento de Personalidad, Evaluación y Tratamientos Psicológicos, Facultad de Psicología, Universitat de València, Valencia, Spain

**Keywords:** obsessive-compulsive disorder, teachers, mental health literacy, brief education, stigmatizing attitudes, stigma, intervention

## Abstract

Because children and adolescents are vulnerable to developing obsessive-compulsive disorder (OCD), classroom teachers play an important role in the early identification and intervention in students with OCD. The present study aims to explore the recognition of OCD, general knowledge about this disorder, implications in the classroom, and stigmatizing attitudes among teachers, as well as the effectiveness of a brief educational intervention about OCD. Participants (*n* = 95; mean age = 43. 29 years old; 64.3% female) were primary and secondary school teachers who were randomly assigned to an experimental group or a control group. All of them completed a set of self-report questionnaires, read an educational fact sheet (either about OCD in the experimental group or about a healthy diet in the control group), and again completed the questionnaires. Results show that prior to the intervention, most of the teachers identified the contamination and order OCD symptoms described in a vignette as specific to OCD (82.1%) and would recommend talking about the problem (98.9%) and seeking help (94.7%). However, only a few (36.8%) knew about the most effective OCD treatments or identified compulsions as a main OCD symptom (33%). Moreover, only about half of the teachers correctly identified OCD's possible interference in classroom routines, such as delays to achieve perfection or concentration problems, and strategies for dealing with OCD, such as continuing with the class rhythm. Stigma levels were from low to moderate. After the brief educational intervention, participants in the experimental group increased their knowledge about OCD, improved their strategies for managing a student with OCD symptoms, and had fewer stigmatizing attitudes associated with pity (*p* < 0.05). These changes were not observed in the control group. We can conclude that this brief and easy-to-administer intervention is an effective educational intervention to significantly improve teachers' knowledge and attitudes, at least in the short-term. These results are especially relevant because OCD is associated with high interference and long delays in seeking treatment, and teachers have a unique opportunity to help with prevention, early identification, and recommending an adequate intervention for OCD.

## Introduction

Obsessive-compulsive disorder (OCD) is considered one of the 10 leading causes of disability worldwide (1), with a lifetime prevalence of 2.30% (National Comorbidity Survey Replication) (2). Children and adolescents are highly vulnerable to developing OCD (3). OCD has been estimated to affect ~1–4% of children and adolescents (4–6), and from 40 to 80% of adults with OCD experienced their first symptomatic manifestations during childhood (7–12). In fact, studies suggest that adult males experience its onset before the age of 10, and adult females during adolescence (2).

Although OCD is associated with high impairment in quality of life (13, 14) and considerable interference in OCD sufferers' lives, including social, emotional, and/or academic functioning (15), there is usually a long delay in seeking treatment in adults (16, 17), as well as in children and adolescents (18, 19). In adults, studies have pointed out that between 38 and 89.8% of OCD sufferers neither ask for nor receive treatment for their symptoms (20–24). This delay in seeking treatment is a serious public health problem that has considerable effects and costs for the individual, family, mental health system, and society (25, 26).

Research suggests that the level of mental health literacy (MHL), that is, the “knowledge and beliefs about mental disorders which aid their recognition, management or prevention” in the community [(27), pp., 182] and the stigma, in other words, “the prejudice and discrimination that robs people with mental illness of opportunities to accomplish personal goals” [(28), pp. 635] associated with OCD would explain the delayed treatment-seeking behavior of OCD patients [e.g., (16, 20, 29)].

Some studies have examined the levels of MHL and public stigma, that is, “the results of the population endorsing the prejudice and discrimination of mental illness” [(28), pp. 635], related to OCD in different populations, such as adolescents (30) or undergraduates [e.g., (31, 32)], parents of children under 18 years old (33), mental health care providers (34), or the general population [e.g., (35–37)]. Primary and secondary teachers play an important role in the detection, referral, and management of OCD symptoms due to their extensive interaction with the students and because children and adolescents, who are vulnerable to developing OCD, spend most of their time in school (38–41). Moreover, primary and secondary teachers often have a limited amount of mental health knowledge (42, 43), do not feel confident about helping students with mental health problems (44), and show stigmatizing attitudes toward mental disorders (45–47). However, little is known about teachers' general knowledge about OCD and its classroom implications. In this regard, only White et al. (48) reported that elementary school teachers had significantly more knowledge about OCD than about Tourette syndrome or attention-deficit/hyperactivity disorder.

The implementation of intervention programs focused on increasing the recognition of OCD, the use of mental health services, and disregarding stigma associated with OCD–and mental disorders in general- could help to eliminate long delays and reduce the personal and financial costs of OCD. In this regard, intervention programs focused on stigma frequently include one or more of the following components: education (replacing the most relevant myths about mental illness with correct information and accurate knowledge), direct or indirect contact (interactions with people who have a mental illness to challenge prejudice), and protest (attempts to suppress stigmatizing attitudes and representations of mental illness) (49). Education has been the most commonly used strategy, and there is evidence that stigma interventions based on educational strategies are effective in reducing stigmatizing attitudes about mental illness and public stigma associated with different mental disorders among members of the community [e.g., (50, 51)]. However, OCD has largely been ignored in the stigma literature (52), and only a few studies have investigated the role of interventions in increasing mental health literacy about OCD and reducing the associated stigma. In general, these interventions were brief and varied in their contents and procedures: reading DSM diagnostic criteria (52, 53) or broader OCD information (54, 55); watching an OCD educational or contact video (56, 57); or short workshops (48). These interventions have been tested in different samples, such as community samples (53, 55), University students (52), clinical, counseling, and school psychologists (56), or school teachers (48, 54). Interventions carried out in schools are especially relevant because OCD often begins in adolescence, and it has been reported that children and adolescents with OCD primarily seek help from general practitioners and school staff (6). In this vein, White et al. (48) examined the effects of a 2-h training workshop about Tourette syndrome, with OCD and attention deficit hyperactivity disorder as possible related conditions, and they evaluated teachers' knowledge before and after the workshop. Results showed an average 5% increase in teachers' knowledge about those disorders. Data on the OCD questions revealed that, on the pretest, participants knew more about OCD than about the other conditions, and there was an unexpected significant decrease in pre- post-test scores on OCD knowledge, which could be due to confusion after increasing the knowledge about the other conditions. The Jassi et al. (54) study was designed to assess the impact of reading an information packet about OCD on the awareness and knowledge of teachers and other school staff about the disorder. Results showed that, after reading the packet, there was an increase in the correct answers on an instrument measuring general knowledge about OCD, its symptoms, and recommended treatment.

The present paper aims to fill the gap in the scientific literature regarding teachers' awareness and attitudes about OCD symptoms, as well as the usefulness of an intervention for school teachers designed to increase their mental health literacy associated with OCD, decrease the stigma, and offer guidelines for necessary actions in working with students with OCD in the classroom. The aims of this study are three-fold: (1) to analyze OCD recognition, general knowledge about OCD (i.e., symptomatology, causes, and treatments), and its implications in the classroom among teachers; (2) to explore OCD public stigma levels in teachers; and (3) to assess the effectiveness of a brief educational intervention for teachers on OCD recognition, general knowledge, implications in the classroom, and stigma associated with OCD.

## Method

### Participants

The sample consisted of 95 teachers from primary and secondary schools from two cities in Spain (Teruel and Valencia). The mean age was 43.29 years (SD = 10.63; ranging from 24 to 65), and the majority were female (64.2%). The majority of the participants were married (51.6%) and had a medium socio-economic level (76.1%), following the parameters of the Spanish National Institute of Statistics. Furthermore, 31.6% of the participants reported having previous contact with mental disorders (including themselves, relatives, or people close to them).

### Instruments

For the present study, an *ad hoc* vignette-based questionnaire was used to assess OCD recognition and treatment recommendations. It includes a vignette describing a 12-year-old boy/girl in a classroom who shows clinically significant contamination/ order OCD symptoms as the most representative of OCD (see Supplementary Material). The vignette was independently evaluated by five clinical psychologists who are experts in OCD to assess its clarity regarding the main diagnosis. After reading the vignette, participants were asked the following questions: (1) Problem recognition [*Is what is happening to Juan/Maria a cause for concern?*-response options: (a) yes, (b) no, (c) undecided-]; If it is a cause for concern, *what do you think is happening to Juan/Maria?*- response options: (a) day-to-day stress, (b) family problems, (c) developmental disorder, (d) school problems, (e) generalized anxiety disorder, (f) schizophrenia, (g) obsessive-compulsive disorder, (h) depression, (i) phobia, (j) personality disorder, (k) medical problems; (2) Perception of causality [*What do you think is the primary cause triggering Juan's/Maria's problem?*; response options: (a) stress, (b) having experienced a trauma, (c) problems at school, (d) discrimination by his/her peers, (e) his/her personality, (f) family problems, (g) influence of friends, (h) virus or nutritional deficiency, (i) influence of television, (j) organic or physical alteration, (k) religious beliefs, (l) personal weakness, (m) mental health problem]; (3) Recommendation to talk about the problem [*Would you recommend that Juan/Maria talk about what is wrong with him/her?*; response options: (a) yes, (b) no, (c) undecided]; (4) Perception of need for treatment [*Do you think Juan/Maria should seek help to solve his/her problem?;* response options: (a) yes, (b) no, (c) undecided]; and (5) Treatment recommendation [*In your opinion, what treatment would be most helpful to Juan/Maria?;* response options: (a) doing leisure activities with his/her parents/family, (b) practicing sports, (c) doing extra-curricular activities, (d) relaxation and stress management courses, (e) meditation or yoga, (f) cognitive-behavioral therapy, (g) psychoanalysis therapy, (h) family therapy, (i) hypnosis]. Participants were asked to choose only one option for each question. These questions and the response options were chosen because they have been shown to be relevant in previous studies that assess MHL associated with OCD and explore the variables associated with the delay in seeking treatment (18, 20, 30, 31, 33, 36, 58). Vignettes describing psychological disorders are frequently used in the stigma and MHL literature [e.g., (27, 32, 59, 60)]. This method offers the opportunity to include a concrete and detailed description of the symptoms experienced by a patient suffering from OCD in his/her context, with the real daily life impairment associated with those symptoms. This description may help participants to think about their attitudes toward the person described or identify whether there is a problem. Moreover, this methodology could be less susceptible to social desirability bias than others (61).

Next, participants were asked to answer the *Attribution Questionnaire-9* [AQ-9; (59)] referring to the vignette. The AQ-9 is a 9-item version of the Attribution Questionnaire- 27 [AQ-27; (62, 63)] that assesses public stigma. The original AQ-9 was developed by selecting the single item that had the highest loading in each factor, showing a Cronbach's alpha of 0.62 in community members and 0.73 in college students (59). The AQ-9 assesses the following stereotypes: pity (feeling sympathy because people are overwhelmed by their illnesses); dangerousness (people with mental illnesses are a threat to others); fear (feeling frightened because people with mental illness are dangerous); blame (people are responsible for their mental illness); segregation (people with mental illness should be removed from their community); anger (feeling irritated or annoyed because people are to blame for their mental illness); help (wanting to assist people with mental illness); and avoidance (staying away from people with mental illness). Research participants respond to individual items (e.g., “How scared of Juan/María would you feel?”) presented in response to a vignette (see Supplementary Material) on a 9-point Likert scale ranging from 1 = “not at all” to 9 = “very much.” The higher the score, the more that factor is being endorsed by the subject. Items on the Spanish version of the AQ-9 were taken from the *Attribution Questionnaire for use in Spanish-speaking populations* [AQ-27-E; (64)], which showed good internal reliability (α = 0.85), similar to what was reported for the original (65) and Italian (66) versions of the instrument. A previous study conducted with the same version of the AQ-9 reported a Cronbach's alpha of 0.65 (67). In the present study, the internal consistency, measured as Cronbach's alpha, was 0.61.

Participants were then asked other questions related to their general knowledge about OCD: (1) Identification of OCD [*OCD is;* response options: (a) a learning disorder, (b) a mental disorder, (c) a set of manias, (d) not sure]; (2) Prevalence of OCD [*The prevalence of OCD in the population is around*; response options: (a) 0.5%, (b) 1–2%, (c) 5–10%, (d) not sure]; (3) Primary symptoms [*The primary symptoms of OCD are* (*more than one alternative is allowed*); response options: (a) obsessions, (b) hallucinations, (c) hobbies, (d) compulsions, (e) autolytic behaviors, (f) not sure]; (4) Definition of obsessions [*Obsessions are;* response options: (a) daily life concerns, (b) hallucinations, (c) repetitive thoughts, images, or impulses, (d) not sure]; (5) Definition of compulsions [*Compulsions are;* response options: (a) repetitive behaviors with the main objective of attracting attention, (b) repetitive behaviors to reduce the discomfort caused by obsessions, (c) unremarkable repetitive behaviors, (d) not sure]; (6) Relationship between intellectual level and OCD [*Is the intellectual level a key factor in the development of OCD?;* response options: (a) yes, OCD is more common in bright children with high skills, (b) yes, OCD is more common in children with learning disabilities, (c) no, OCD affects children with different abilities, (d) no, OCD is more related to the child having behavior problems]. Finally participants were asked about possible implications of OCD in the classroom: (7) OCD signs that are observable in the classroom [*Some signs of OCD that are observable in the classroom would be* (*more than one alternative is allowed*); response options: (a) social isolation, (b) delay in finishing tasks to seek perfection, (c) low weight, (d) anxiety about changing routines, (e) exaggerated desire to be a leader, (f) comprehension problems, (g) concentration problems, (h) tires quickly of routines and needs new stimuli, (i) spends a lot of time ordering objects]; and (8) how to deal with a student with OCD who is performing a compulsion [*When a child with possible OCD is performing a ritual or compulsion* (*more than one alternative is allowed*); response options: (a) attempt to convince the child to stop the ritual, (b) make him/her see that his/her concern is absurd, (c) continue with the rhythm of the class, (d) because it is sometimes an almost automatic behavior, you should call his/her attention to stop it, (e) he/she will be given more time to turn in assignments]. Participants could choose one or more option for questions 3, 7, and 8.

### Procedure

Teachers from six Spanish primary and secondary schools were invited to participate in an investigation on health-related variables. Schools were chosen by convenience. First, one of the authors contacted the head of the school and asked for the school's participation in the research study. After obtaining his/her agreement, teachers were invited to participate by e-mail. Interested participants were randomly assigned to either a control or an experimental group with a 1:1 allocation according to a computer-generated preset randomization list (randomizer.org). Next, participants received the instruments and material in person, and they were asked to answer the questionnaires described above and then read a fact sheet about OCD characteristics (experimental group) or about diet (control group). After reading the fact sheet, all the participants completed the questionnaires again. All the participants completed an informed consent form, and the study was approved by the University Ethics committee.

### Materials

#### OCD Educational Intervention

It consists of reading a fact sheet about OCD, specifically the definition of the disorder, obsessions and compulsions, its prevalence, recommended treatment, where to access professional support, keys to detecting the disorder in the classroom, and some basic advice on how to act with affected students.

#### Healthy Diet Educational Intervention

It consists of reading a fact sheet that includes general information about how to eat a healthy diet. Specifically, it contains information about the basic principles of healthy eating, an explanation of what healthy food should be, a list including of healthy food and necessary nutrients, and some tips to encourage healthy eating habits. It does not provide any information about OCD. Both fact sheets presented a similar structure in terms of their organization on two sheets divided into two columns and their length (630–650 words).

### Analysis

Descriptive statistics were used to report frequencies, means, and standard deviations. Categorical variables were transformed into dichotomous variables (i.e., number of people who chose the correct option vs. the number of people who chose any other option) to facilitate interpretation of the results. On questions with more than one correct answer, each correct option was considered. Participants were grouped depending on the intervention received: experimental group (*n* = 49), where they had to read an OCD fact sheet, and control group (*n* = 46), where participants had to read a healthy diet fact sheet. McNemar's-test was used to compare related groups (pre- and post-intervention) on categorical dichotomous MHL variables. A repeated-measures Wilcoxon non-parametric test was used to compare quantitative data (AQ-9). All analyses were conducted using SPSS 25.

## Results

### Mental Health Literacy and Stigma Associated With OCD

Regarding the level of OCD recognition and treatment recommendations, after reading the vignette, all the teachers recognized that there was a problem, and most of them identified the symptoms described as specific to OCD (82.1%), although slightly less than half (46.3%) of the teachers identified the problem described as a mental health problem. Nevertheless, most of the teachers would recommend that the adolescent talk about the problem (98.9%) and seek help (94.7%). However, only a small proportion of teachers (36.8%) reported that cognitive-behavioral therapy would be the most helpful treatment for the student described (see Table 1).

**Table 1:** Descriptive statistics of OCD recognition and treatment, general knowledge about OCD and its implications in the classroom and the stigma related to OCD, and comparison of these variables before (pre) and after (post) the intervention in the experimental and control groups.

	**Item**	**Total sample (*N* = 95)**	**Experimental group (*****n*** **=** **49)**			**Control group (*****n*** **=** **46)**		
		**Pre**	**Pre**	**Post**	***p***	**Pre**	**Post**	***p***
OCD recognition and	Problem recognition	100 (95)	100 (49)	100 (49)	–	100 (46)	100 (46)	–
treatment	Problem recognition: *OCD*	82.1 (78)	79.6 (39)	93.9 (46)	0.039	84.8 (39)	80.4 (37)	0.625
recommendation (MHL)	Causality*: mental health problem*	46.3 (44)	49 (24)	63.3 (31)	0.039	43.5 (20)	47.8 (22)	0.727
	Recommendation to talk	98.9 (94)	98 (48)	100 (49)	–	100 (46)	100 (46)	–
	Need for treatment	94.7 (90)	95.9 (47)	100 (49)	–	95.6 (43)	100 (46)	–
	Treatment recommendation: *CBT*	36.8 (35)	36.7 (18)	71.4 (35)	0.000	37 (17)	32.6 (15)	0.625
General knowledge	OCD*: mental disorder*	58.5 (55)	60.4 (29)	83.7 (41)	0.007	56.5 (26)	62.2 (28)	0.250
about OCD (MHL)	Prevalence: *1–2%*	16.8 (16)	10.4 (5)	79.6 (39)	0.000	23.9 (11)	21.7 (10)	1
	**Primary symptoms:**							
	*Obsessions*	78.7 (74)	75 (36)	95.9 (47)	0.006	82.6 (38)	84.8 (39)	1
	*Compulsions*	33 (31)	39.6 (19)	91.8 (45)	0.000	26.1 (12)	39.1 (18)	0.109
	Definition of obsessions	73.4 (69)	70.8 (34)	87.8 (43)	0.039	76.1 (35)	69.6 (32)	0.453
	Definition of compulsions	78.7 (74)	79.6 (39)	95.9 (47)	0.021	77.8 (35)	82.6 (38)	0.500
	Intellectual level: *not a key*	64.8 (35)	69 (20)	79.3 (23)	0.375	60 (15)	69.2 (18)	0.625
Implications of OCD in the classroom (MHL)	**Observable classroom signs:**							
	*Social isolation*	61.7 (58)	59.2 (29)	63.3 (31)	0.815	64.4 (29)	60.9 (28)	0.687
	*Delay to achieve perfection*	58.5 (55)	59.2 (29)	79.6 (39)	0.013	57.8 (26)	58.7 (27)	1
	*Anxiety about change*	76.6 (72)	71.4 (35)	75.5 (37)	0.791	82.2 (37)	71.7 (33)	0.180
	*Concentration problems*	47.9 (45)	44.9 (22)	59.2 (29)	0.118	51.1 (23)	52.2 (24)	1
	*Spends time ordering*	72.3 (68)	77.6 (38)	77.6 (38)	1	66.7 (30)	67.4 (31)	1
	**How to deal with compulsions in the classroom:**							
	*Continue class rhythm*	48.9 (46)	49 (24)	85.7 (42)	0.000	48.9 (22)	48.9 (22)	1
	*Giving more time*	61.7 (58)	53.1 (26)	83.7 (41)	0.000	71.1 (32)	63 (29)	0.453
Stigma (AQ-9)	*Pity*	3.81 ± 2.53	4 ± 2.51	3.16 ± 2.53	0.004	3.60 ± 2.58	3.40 ± 0.68	0.146
	*Dangerousness*	2.69 ± 2.20	2.60 ± 2.38	2.39 ± 2.14	0.455	2.78 ± 2.02	2.02 ± 1.63	0.006
	*Fear*	2.58 ± 2.02	2.38 ± 2.02	2.12 ± 1.87	0.145	2.80 ± 2.01	1.40 ± 1.23	0.000
	*Blame*	1.63 ± 1.30	1.77 ± 1.43	1.41 ± 0.91	0.064	1.49 ± 1.16	1.11 ± 0.48	0.518
	*Segregation*	1.25 ± 0.88	1.36 ± 1.13	1.29 ± 1.33	0.498	1.13 ± 0.50	1.16 ± 0.42	0.317
	*Anger*	1.28 ± 0.63	1.21 ± 0.50	1.10 ± 0.36	0.160	1.36 ± 0.74	7.61 ± 1.58	0.059
	*Help*	7.56 ± 1.55	7.48 ± 1.54	7.96 ± 1.55	0.006	7.65 ± 1.58	7.61 ± 1.58	0.617
	*Avoidance*	1.26 ± 0.87	1.13 ± 0.39	1.27 ± 1.05	0.518	1.40 ± 1.17	1.20 ± 0.62	0.131
	*Coercion*	5.98 ± 2.57	6.04 ± 2.69	6.22 ± 2.65	0.239	5.91 ± 2.47	5.64 ± 2.69	0.243

*OCD, obsessive-compulsive disorder; MHL, mental health literacy; AQ-9, Attribution Questionnaire-9*.

*Data on pre- and post-columns are expressed as % (n) of participants giving the correct answer or as M ± SD*.

When teachers were asked about their general knowledge about OCD, just over half of them identified OCD as a mental illness (58.5%), and in a few cases its prevalence was correctly identified as between 1 and 2% (16.8%). The majority of the teachers identified obsessions as a primary symptom of OCD (78.7%), but only a small portion of them identified compulsions as a primary symptom (33%). Nonetheless, most of the participants correctly identified the definition of obsession (73.4%) and compulsion (78.7%). Finally, 64.8% correctly identified that OCD affected children regardless of their intellectual level.

Regarding OCD's implications in the classroom, between 47.9% (concentration problems) and 76.6% (anxiety about changing routines) of the teachers properly identified possible interference of OCD in classroom routines. In addition, between 48.9% (continue with the rhythm of the class) and 61.7% (he/she will be given more time to turn in assignments) of the teachers selected appropriate patterns of behavior if a student was performing a compulsion.

Stigma levels, measured by the AQ-9, were between low and moderate, ranging between 1.25 (*segregation*) and 7.56 (*help*) on a scale from 1 to 9. As Table 1 shows, *help* was the construct with the highest score, followed by *coercion* and *pity*. The constructs with the lowest scores were *blame, anger, avoidance*, and *segregation*.

### Effects of a Brief Intervention on MHL and OCD-Related Stigma

To determine whether the intervention was effective, pre- and post-intervention scores of the experimental (*n* = 49) and control (*n* = 46) groups were compared using the McNemar and Wilcoxon tests. The two established groups (experimental vs. control) did not significantly differ on the sociodemographic variables, previous experience with mental disorders, or the MHL and OCD-related stigma variables (*p* > 0.005). After the intervention (reading the OCD fact sheet), participants in the experimental group increased their knowledge about most of the MHL variables measured (12 out of 20), and on three variables, there was no change because participants previously knew the correct answer (see Table 1). Specifically, regarding *OCD recognition and treatment*, a significantly higher percentage of participants correctly identified the vignette as describing OCD, indicated that mental health was the cause of the problem, and selected cognitive-behavioral therapy as the reference treatment for OCD. In contrast, no differences were found in the control group before and after reading the healthy diet fact sheet. Regarding *general knowledge about OCD*, significant differences were found in the experimental group on: the recognition of OCD as a mental disorder, the estimation of prevalence at about 1–2%, the recognition of obsessions and compulsions as the primary symptoms of OCD, and the correct definition of obsessions and compulsions. With regard to the *implications of OCD in the classroom*, significant differences were observed in identifying the delay in finishing tasks to seek perfection as a consequence of OCD, and in identifying the two variables associated with dealing with an OCD student who is performing a compulsion, i.e., continuing with the class schedule and giving more leeway in turning in tasks. No differences were found in the control group.

In the case of stigma, after the intervention, in the experimental group, scores on the *pity* variable were lower, and scores on willingness to *help* were higher, whereas in the control group, there was a decrease in the scores on *dangerousness* and *fear* (see Table 1).

## Discussion

This is the first study to explore teachers' knowledge, identification, and attitudes about OCD and evaluate the effectiveness of a brief intervention about OCD in an educational context, including a control group. Specifically, this study aims to better understand the perceptions and attitudes about OCD among teachers because a lack of knowledge and skills or stigmatizing attitudes toward OCD are barriers that would keep teachers from supporting their students with mental health problems. Results show that teachers identify the vignette symptoms as part of OCD and would recommend that adolescents and children talk about the problem and seek help. These data are very positive and consistent with previous studies showing a high percentage of identification of OCD in vignettes describing contamination or order/symmetry OCD symptoms in undergraduates (31) or adolescents (30), and a high recommendation to seek help [e.g., (36)]. However, teachers failed to view the problem described in the vignette as a mental health problem, and they had poor knowledge about evidence-based treatments. Moreover, although most of the participants correctly identified the definitions of obsession and compulsion, when they were asked specifically about OCD, they did not identify it as a mental health problem or consider compulsions to be relevant as a primary symptom of the disorder. Teachers seem to identify OCD more with obsessions than with compulsions. This result is not consistent with a recent study that reported that community participants identified OCD more with compulsions than with obsessions (37). Finally, teachers refer to having scarce skills for managing their students' OCD symptoms in the classroom. Thus, only a few teachers selected appropriate steps for action when a student is performing a compulsion, and most of them chose the wrong strategies to deal with OCD symptoms in class, which could increase the child's anxiety and discomfort. Therefore, the results clearly support the need to initiate intervention programs in the educational context.

The second aim of the present study was to explore stigma levels associated with OCD in teachers. Teachers showed low levels of stigma, and they reported that they would not avoid students, get angry with them, or blame them for their OCD symptoms. Nor do teachers think students with OCD should be confined to a psychiatric hospital. Moreover, these students would not inspire pity or fear or be considered dangerous. Furthermore, teachers showed a high tendency to help students with OCD. Finally, the teachers believed that students with OCD should get medical treatment, even if they refuse it (coercion). The data are quite similar to those reported by adolescents for ordering OCD (30), or by adults when observing symmetry and contamination vignettes (68) or watching a video of patients reporting not just right obsessions (69).

Finally, we also aimed to test the effectiveness of a brief educational intervention designed to improve teachers' knowledge, skills, and attitudes toward OCD. Results showed that reading the educational intervention about OCD (but not reading the healthy diet educational information) had a positive impact by increasing their knowledge about almost all the variables evaluated where improvement was possible. The intervention led teachers to identify that there was a mental health problem, and that cognitive-behavioral therapy was the reference treatment for OCD. This result is especially relevant because adolescent students are reluctant to seek professional help (18, 19), and ineffective experiences with treatment are a barrier to seeking help (70). Moreover, teachers learned that OCD is a mental health disorder, and they learned about its prevalence, main symptoms, and how to deal with students showing compulsions in the classroom. This knowledge will provide teachers with strategies for detecting students with possible difficulties. Moreover, when dealing with diagnosed students, teachers will have some tools to manage them without interfering with the problem, and they will be able to avoid dysfunctional strategies. However, although teachers' knowledge improved considerably, the intervention did not improve the identification of all the difficulties OCD can cause in classroom, perhaps because this information was presented in a compact way and without examples. Future studies should improve this part of the intervention because it is highly relevant. In general, the results are consistent with the Jassi et al. (54) study, which reported an improvement in OCD knowledge in school staff, and with other brief interventions in community participants showing that just reading the OCD diagnostic criteria decreases negative attitudes (52, 53).

After the intervention, *Pity*, one of the previously most highly stigmatizing attitudes, was lower, probably due to the increase in OCD literacy. At the same time, there was an increase in *Help*. This result is striking because, following the attribution theory, these pro-social attitudes develop when individuals are thought to have little responsibility or control over their condition (71). However, in this context, where teachers are asked how likely it is that they would help one of their students who showed interfering symptoms, help could be understood as the empowerment of teachers who, after improving their knowledge, feel that they can do something more to manage the symptoms in the classroom. It was surprising that in the control group there was also a significant decrease in two of the stigmatizing attitudes: dangerousness and fear. Although we do not know the reason for this decrease without any intervention, we can hypothesize that participants were merely influenced by reading a vignette about OCD twice and answering related questions. Moreover, the fact that participants felt observed and tested may have influenced the decrease in these two stigmatizing variables. According to the Hawthorne effect, awareness of being observed or having one's behavior assessed engenders beliefs about researcher expectations (72). Future studies should include a measure of social desirability because measures like the AQ could be influenced by social desirability.

In general, the results show that the educational strategy not only improves the knowledge about the disorder and empirical-based treatments, but it also offers strategies for managing OCD symptoms in the classroom, allowing teachers to feel empowered. Moreover, this brief educational sheet seems to change public stigma, or at least behavioral intentions as a proxy for behavior. However, the results are probably not only a result of the educational strategy, but they might also be influenced by reading the vignette twice, in the pre-test and at the beginning of the post-test. Reading the vignette allowed teachers to put the symptoms and interference described in the educational sheet into context and connect with the difficulties and suffering of a fictional student suffering from OCD obsessions and compulsions. Although we developed an educational intervention, including the vignette added a kind of “indirect contact” strategy that probably improved or strengthened the effect of the educational intervention.

Despite the positive results of the study, there are some limitations that we would like to mention. First, we chose a vignette representing contamination and order obsessions and compulsions as the most representative of OCD because they are the most prevalent presentations in OCD patients (17, 73) and the most popular in social media such as television series or movies (74). However, this decision has the disadvantage that general population is more familiar with these kinds of obsessions, and so it is reasonable that this familiarity might influence OCD literacy levels and stigmatizing attitudes. Would we have found the same results with a vignette with sexual or aggressive content? Probably not. Previous research has shown in different samples (i.e., students, adults, or even clinicians) that OCD contamination and order/ symmetry modalities are recognized more than others (30, 34, 52, 68), and that sexual or aggressive thoughts are associated with higher stigmatizing attitudes, social distance, and rejection than other OCD modalities [e.g., (30, 60, 68, 74)]. Future research should assess the effectiveness of intervention programs that present OCD as a heterogeneous disorder. Although we described this heterogeneity in the brief intervention, we did not assess differences between obsessional contents. Moreover, a possible limitation of the assessment of stigmatizing attitudes is the low reliability coefficient for the AQ-9, a limitation that we tried to overcome by using the instrument at the item level and not as a total score. Another limitation is the lack of a follow-up, which means that we are unable to determine whether the changes are maintained over time. Therefore, future studies should measure the stability of the changes. Moreover, the convenience sample recruited in this study limits the generalizability of the findings, and the fact that only interested teachers participated could have influenced the positive results of the intervention. Future studies should also further address OCD interference in the classroom, and the program could be improved by providing the information through a mental health application or a website to make it easier to access.

We can conclude that the present brief educational intervention improves teachers' knowledge and attitudes significantly, at last in the short term. These types of programs are especially relevant because OCD is associated with high interference and long delays in seeking treatment. Teachers, who are in daily contact with students who could be developing OCD, have a unique opportunity to prevent OCD development by helping with early identification and adequate interventions for OCD. Moreover, they could promote positive attitudes toward OCD among their students. In fact, higher rates of peer victimization have been reported in youth with OCD than in healthy controls and children with Type I diabetes, and these high rates are associated with severity and depression (75). Longer interventions and other modalities such as workshops offer the opportunity to provide more information and include other mechanisms apart from psychoeducation, such as direct/indirect contact or cognitive restructuring. However, by using this brief, easy-to-administer (the use of information sheets), and effective intervention, we are able to reach more teachers. Instead of a program training teacher to be experts in OCD, it seems preferable to offer a lot of teachers some notions about OCD that could aid in its prevention and early identification. Moreover, these kinds of programs are easily distributed over the Internet to the population of interest, in this case teachers, which would be preferable to face-to-face formats in a situation such as the current COVID-19 pandemic. Through the widespread use of these kinds of easy-to-administer interventions, we can avoid unnecessary suffering in children and adolescents with OCD symptomatology. Their teachers (adults of reference where they spend most of their time) could provide early detection and recommend that they ask for formal help, which would lead to more effective therapeutic outcomes. Moreover, teachers could adequately manage symptoms in the classroom, and victimization of young OCD patients by their peers could be reduced.

## Data Availability Statement

The raw data supporting the conclusions of this article will be made available by the authors, without undue reservation.

## Ethics Statement

The studies involving human participants were reviewed and approved by Comité Ético de Investigación en Humanos de la Comisión Ética en Investigación Experimental de la Universitat de València. The patients/participants provided their written informed consent to participate in this study.

## Author Contributions

GG-S and MR contributed to conception, design, data collection of the study, and conducted the manuscript revision. SA organized the database, performed the statistical analysis, and wrote the first draft of the methods and results sections. AC wrote the first draft of the introduction and discussion sections. All authors revised, read, and approved the submitted version.

## Conflict of Interest

The authors declare that the research was conducted in the absence of any commercial or financial relationships that could be construed as a potential conflict of interest.
