# Supplementary material for: Teachers' Knowledge and Stigmatizing Attitudes Associated With Obsessive-Compulsive Disorder: Effectiveness of a Brief Educational Intervention
Source: Front Psychiatry. 2021 Jun 2;12:677567. doi: 10.3389/fpsyt.2021.677567 (PMC8206535; doi:10.3389/fpsyt.2021.677567)
Supplement: Supplementary file 1 [file Table_1.DOCX]

Vignette.

María/ Juan is a 12-year-old girl/boy who attends the classroom where you teach. In the past few months, she/he has been overly concerned about cleanliness, and you have noticed that she/he spends much more time than her/his classmates washing her/her hands. Every time she/he touches something her/his classmates have touched (e.g., a book), she/he tries to go to the toilet to wash her/ his hands, which she/he does 5 times in a row. If you don't let her/him go to the bathroom to wash her/his hands, she/he gets very nervous and worries during the rest of the class until she/he can wash her/his hands. You have also noticed that when she/he enters the classroom, she/he avoids touching the door with her/his hands and tries to do so with her/his jumper or a tissue. She/he has stopped eating in the school canteen. Her/his parents say that she/he can't stand eating there because she/he says she/he could become contaminated by using the cutlery in the dining room.

In addition, you have noticed that in the classroom, she/he needs to have her/his desk perfectly positioned. She/he cannot stand it if the desk is not aligned with the lines of the floor tiles, and everything on her/his desk has to be in a position that she/he considers correct (e.g., her/his worksheets and books aligned, pencil case on the right). Also, you have noticed that every time she/he sits down in the classroom, she/he jumps forward three times and then backwards three times. When you tell her/him not to do that, María/Juan becomes very nervous and cries. At the end of the lesson, she/he explains to you that if she/he doesn’t jump up and down, her/his parents might have a car accident.
